# Seasonal and Spatial Variations of Heavy Metals in Two Typical Chinese Rivers: Concentrations, Environmental Risks, and Possible Sources

**DOI:** 10.3390/ijerph111111860

**Published:** 2014-11-17

**Authors:** Hong Yao, Xin Qian, Hailong Gao, Yulei Wang, Bisheng Xia

**Affiliations:** 1 State Key Laboratory of Pollution Control and Resource Reuse, School of the Environment, Nanjing University, Nanjing 210023, China; E-Mails: yaohong80@126.com (H.Y.); njughl@gmail.com (H.G.); gywyl127@163.com (Y.W.); xiabisheng1984@163.com (B.X.); 2 School of Geography, Nantong University, Nantong 226001, China

**Keywords:** heavy metals, seasonal and spatial variations, risk assessment, source apportionment, risk prevention and mitigation

## Abstract

Ten metals were analyzed in samples collected in three seasons (the dry season, the early rainy season, and the late rainy season) from two rivers in China. No observed toxic effect concentrations were used to estimate the risks. The possible sources of the metals in each season, and the dominant source(s) at each site, were assessed using principal components analysis. The metal concentrations in the area studied were found, using *t*-tests, to vary both seasonally and spatially (*P* = 0.05). The potential risks in different seasons decreased in the order: early rainy season > dry season > late rainy season, and Cd was the dominant contributor to the total risks associated with heavy metal pollution in the two rivers. The high population and industrial site densities in the Taihu basin have had negative influences on the two rivers. The river that is used as a source of drinking water (the Taipu River) had a low average level of risks caused by the metals. Metals accumulated in environmental media were the main possible sources in the dry season, and emissions from mechanical manufacturing enterprises were the main possible sources in the rainy season. The river in the industrial area (the Wusong River) had a moderate level of risk caused by the metals, and the main sources were industrial emissions. The seasonal and spatial distributions of the heavy metals mean that risk prevention and mitigation measures should be targeted taking these variations into account.

## 1. Introduction

Heavy metals are important pollutants in surface waters, causing persistent environmental hazards that can seriously harm human and ecological health [1,2,3,4]. Heavy metals in surface waters originate from natural processes, such as atmospheric deposition and geological weathering, and from anthropogenic activities (in emissions such as industrial wastewater and domestic sewage). The contributions of these sources are different in different regions and in different seasons, so heavy metal concentrations in surface water can vary both spatially and seasonally. Information on these variations is important for decision makers involved in environmental risk management [5,6,7,8,9].

Heavy metals are present in surface water in various forms, which can be classified as soluble (compounds or free ions) and particulate (colloidal or adsorbed to suspended solids). Different forms of the metals exhibit different biological toxicities and environmental behaviors. The free (hydrated) ions of many metals cause chronic toxicity in aquatic organisms [2,3]. Suspended solids are the dominant carriers of heavy metals in surface waters, and are responsible for 60%–97% of the total metal concentrations [10,11,12,13,14]. There are constant dynamic fluxes between the dissolved ion and particulate-bound forms of heavy metals [15],and metals in the soluble and particulate-bound forms are both crucial to the levels of environmental risk that the metals pose. The total metal concentration (*i.e.*, the sum of the concentrations of the dissolved and particulate-bound forms) can be used to represent the overall and long-term risks associated with heavy metal contamination in a well-mixed surface water body [16].

The Taihu basin is one of the most developed regions of China, and large amounts of wastewater containing heavy metals are discharged into surface waters in the basin every year [17]. The rainy season in this area, when there are almost continuously overcast skies and rainy weather, usually lasts for about one month, and is in June and July. The objectives of the study presented here were: (1) to determine the concentrations of a range of heavy metals in the surface water in two typical rivers in the Taihu basin, in areas with different land-use characteristics; (2) to assess the potential environmental risks associated with the heavy metal concentrations in the rivers, including assessing spatial and seasonal variations and determining the dominant metal types in terms of their contributions to the overall risk; (3) to attempt to determine the sources of the metals and to identify the dominant sources at different sites and in different seasons, to provide appropriate suggestions for the effective prevention and mitigation of risks associated with heavy metals in the Taihu basin.

## 2. Methods and Materials 

### 2.1. Study Area

**Figure 1:**
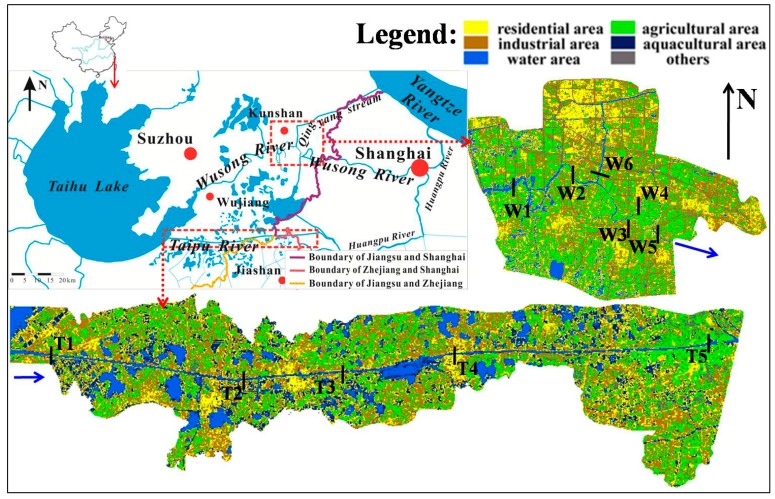
Locations of the study area and observed sites.

The Taipu River and the Wusong River were studied. Both rivers flow out of Taihu Lake in the eastern part of the Taihu basin, and run through a number of provinces (Figure 1). The Taipu River is protected so that it can be used to supply drinking water, and the downstream part of the river is used to supply drinking water to Shanghai. The upstream part of the river is in Wujiang, which is densely populated and has a lot of economic activities.

There are 95 centralized sewage disposal sites along the Taipu River, and there are also industrial sites, mostly dedicated to the manufacture of machinery [18]. The other area that was studied was the section of the Wusong River in Kunshan, which is, economically, the strongest county in China. The Qingyang stream, which is the main tributary of the Wusong River, runs through the urban part of the city of Kunshan (Figure 1). The Wusong River mainly serves industry, and there are more than 300 industrial sites along the section that was studied. Chemical production, textile manufacturing, and printed circuit board production are the dominant industries [18].

### 2.2. Sampling

Taking into account the distribution of potential pollution sources, 11 sampling sites were selected, five (sites T1–T5) on the Taipu River and six (sites W1–W6) on the Wusong River (Figure 1). The longitudes and latitudes of the eleven sites are (120.50750° E, 31.00901° N)-T1, (120.61377° E, 30.99908° N)-T2, (120.73317° E, 31.00129° N)-T3, (120.83725° E, 31.01663° N)-T4, (121.04840° E, 31.02662° N)-T5, (120.88905° E, 31.31697° N)-W1, (120.95352° E, 31.32518° N)-W2, (121.02087° E, 31.29885° N)-W3, (121.01434° E, 31.28713° N)-W4, (121.04413° E, 31.27504° N)-W5, (120.97993° E, 31.33066° N)-W6. Site T1 was about 3 km from Taihu Lake, and was used to represent the outflow of the lake. Site T5, which was in Shanghai, was chosen to represent the water quality in the downstream part of the river. Similarly, site W1 was used to represent the outflow from the upper reaches of the Wusong River and site W5 was chosen to represent the pollutant load emitted in Jiangsu province and reaching Shanghai through the Wusong River. Site W6 was in a tributary of the Wusong River. 

Samples were collected during three periods, the first ones was after two weeks of sunny days in April 2013, the other at the fourth day of the rainy season (in June), and the rest at the last day of the rainy season (in July) and in total 33 samples were obtained. The three batches of samples were assumed to represent the characteristics of the surface water in the rivers in the dry season, early in the rainy season, and late in the rainy season, respectively. The samples were each taken from a bridge using a 5 L Plexiglas sampler. The samples were collected from 20 cm below the water surface. Three 10 L samples were collected at each sampling site, one from the midpoint of the river and one from each of the positions midway between the midpoint and the banks. The samples from a sampling site were immediately mixed together, and a 1 L subsample was taken in a polytetrafluoroethylene bottle that had been soaked in dilute nitric acid for 48 h. The samples were preserved, transported to the laboratory at 4 °C, and analyzed as soon as possible.

### 2.3. Analytical Methods and Quality Control

Four 25 mL subsamples were taken from a sample and mixed together, then this composite sample was digested following the US Environmental Protection Agency (EPA) method 3010 for the acid digestion of aqueous samples and extracts for total metal analysis by inductively couple plasma spectroscopy [19]. All of the vessels used were soaked in 50% nitric acid_(aq)_ for 24 h before use. Concentrated nitric acid (5 mL) was added to each 100 mL water sample, and the sample was evaporated to a small volume (near dryness) at below its boiling point (at 95 °C). The concentrated sample was then digested with concentrated nitric acid, hydrochloric acid, and hydrogen peroxide, and again evaporated to near dryness at 95 °C. The sample volume was then adjusted to 10 mL. Finally the heavy metal concentrations in the sample were analyzed using an inductively coupled plasma atomic emission spectroscopy instrument.

There are no standardized reference materials for surface water, so a soil matrix standard sample (ESS-2) and the standard substance used for analyzing stream sediment compositions (GSD-14), both provided by the Chinese national standards center, were dispersed in deionized water to form a suspension that was used to emulate a standardized water reference material, to test the validity of our method. The relative standard deviations for the elements that we measured were 0.5%–8.2%, and the recoveries were 86%–107%. The precision and accuracy achieved were within the range required for the method to be considered valid. Blanks were analyzed in each batch of samples throughout the entire analytical procedure.

### 2.4. Risk Assessment

In environmental risk assessments, the risk ratio is usually calculated using no observed toxic effects concentrations (NOECs) as the baseline for potential hazards from metals in aquatic ecosystems [20,21,22]. The comprehensive risk index (CRI) is defined as the sum of the risk ratios, and we used it to evaluate the total risk posed by all of the metals we analyzed. The risk ratio for metal *i* (R*_i_*) and the CRI were calculated using the following formulae:

R*_i_* = T*_i_* × OC*_i_*/NOEC*_i_* and (1)

(2) $$\text{CRI}=\sum_{i=1}^{n}R_{i}$$

where OC*_i_* is the observed concentration of metal *i*, and NOEC*_i_* is the highest concentration of metal *i* that does not affect an aquatic ecosystem. The dose of the dissolved metal that will lead to toxic effects can be used to reflect the relative risk from the total metal dose, and so dissolved metal toxicities were used in the R*_i_* calculations, because NOECs for total metal concentrations are unavailable. The risk ratios obtained in this study were, therefore, the relative risks posed by the total metal concentrations rather than the absolute risks posed. An evaluation endpoint using *Daphnia magna*, which is an aquatic organism that is frequently used for analyzing risks in surface water, was selected, and the literature was searched for the NOECs for the heavy metals we studied for this organism [23]. T*_i_* is the toxicity coefficient of metal *i*, which could mirror the potential hazard posed by a metal to the aquatic ecosystem. The T*_i_* values were taken from available publications [16,20,21,22], and were 1 for Ba, 30 for Cd, 2 for Cr, 5 for Cu, 1 for Fe, 1 for Mn, 5 for Ni, 5 for Pb, 2 for V, and 1 for Zn.

No potential environmental risk was expected if R*_i_* < 1, 1 ≤ R*_i_* < 40 was taken to indicate that metal *i* posed a low level of potential environmental risk, 40 ≤ R*_i_* < 80 was taken to indicate moderate risk, 80 ≤ R*_i_* < 160 to indicate considerable risk, 160 ≤ R*_i_* < 320 to indicate a high level of risk, and R*_i_* ≥ 320 was taken to indicate a very high level of risk [16,20,21,22].The classification criteria that should be used for the comprehensive risk index depend on the number of metals involved in the assessment and the sensitivity of the aquatic ecosystem to the metals [16,17,22]. Based on the number of metals we measured, a CRI grading scale that was previously determined mainly using the natural background metal concentrations in soil in the Taihu basin [17] was used. A CRI < 60 was taken to indicate that the metals in the surface water posed low levels of potential environmental risk, 60 ≤ CRI < 120 was taken to indicate moderate risk, 120 ≤ CRI < 240 to indicate a high level of risk, and a CRI ≥ 240 to indicate very high levels of environmental risk.

### 2.5. Source Analysis

Principal components analysis (PCA) was used to identify the potential sources of the heavy metals. The PCA model used is a traditional method for source apportionment, and it can summarize data that have many variables, giving a smaller set of synthetic composite variables [24,25,26,27,28,29]. A detailed description of the PCA procedure can be found elsewhere [26,29].

## 3. Results and Discussion

### 3.1. Heavy Metal Concentrations 

#### 3.1.1. Average Heavy Metal Concentrations in the Rivers

The concentrations of the ten metals that were analyzed (Ba, Cd, Cr, Cu, Fe, Mn, Ni, Pb, V, and Zn) in the two rivers that flow out of Taihu Lake are listed in Table 1. The average Fe, Mn, and Pb concentrations were higher than the drinking water standards recommended by the World Health Organization and the USEPA and listed in Table 2 [30,31]. The metal concentrations were much higher in both rivers than in Taihu Lake (Table 2), and the Cu and Pb concentrations were several times higher in the rivers than in the lake (Cu concentrations of about 4 µg/L and Pb concentrations of about 17 µg/L have been found in the lake [32]). This indicates that the dense population and high number of industrial sites in the Taihu basin could have had negative influences on the metal concentrations in the surface water in the basin, not only in industrial areas but also in the area that is protected so that it can be used to supply drinking water. We compared our results with other reports of metal concentrations in water, and found that most of the metals were present at higher concentrations in the Taipu River than in water used to supply drinking water elsewhere [33,34,35,36], and the Cu, Fe, Mn, Ni, V, Pb, and Zn concentrations were mostly higher in the Wusong River than in polluted industrial areas elsewhere [28,37,38,39,40,41].

#### 3.1.2. Seasonal and Spatial Variations in Metal Concentrations

The metal concentrations were 1.2–2.0 times higher in the industrial area than in the area that is protected for supplying drinking water. The average Ni and Pb concentrations were statistically significantly higher (determined using independent samples *t*-tests; *P* = 0.05) in the Wusong River than in the Taipu River in the dry season. The Cd, Fe, Ni, Pb, and V concentrations in the early rainy season and the Cu, Fe, Ni, and Pb concentrations in the late rainy season were also significantly higher in Wusong River than in the Taipu River (*P* = 0.05). The statistically higher values are shown in bold in Table 1.

In general, the metal concentrations were the highest in the early rainy season, next highest in the dry season, and lowest in the late rainy season. The Ba, Cr, Cu, Mn, Ni, and Zn concentrations in the Taipu River and all of the metal concentrations in the Wusong River were statistically significantly higher in the early rainy season than in the other two seasons (*P* = 0.05). The metal concentrations in the Taipu River were 1.1–3.0 times higher in the early rainy season than in the dry season, with the Cu, Mn, and Zn concentrations being more than two times higher in the early rainy season than in the dry season.The average metal concentrations in the Wusong River were 1.7–5.2 times higher in the early rainy season than in the dry season, with the Cr, Cu, Fe, Pb, V, and Zn concentrations being more than two times higher in the early rainy season than in the dry season. These temporal variations in the metal concentrations were generally consistent with seasonal variations in metal concentrations that have been found in other rivers [6,7].

**Table 1:** Heavy metal concentrations in water samples from the Taipu River and the Wusong River in different seasons; units: ug/L.

Rivers	Seasons	*C*	Ba	Cd	Cr	Cu	Fe	Mn	Ni	Pb	V	Zn
T.R.	DS	Range	72–92	1–5	7–12	10–34	194–472	15–130	13–19	40–63	5–7	39–114
		Avg.± SD	80 ± 10	3 ± 2	9 ± 2	16 ± 10	334 ± 108	97 ± 49	16 ± 2	57 ± 9	6 ± 1	64 ± 31
	ER	Range	140–170	1–5	13–19	20–88	202–636	98–410	13–29	30–188	7–10	120–240
		Avg.± SD	158 ± 13	3 ± 2	17 ± 3	43 ± 26	423 ± 166	266 ± 119	24 ± 7	108 ± 71	9 ± 1	190 ± 47
	LR	Range	70–80	0	0	13–22	96–300	70–210	2–6	7–9	15–20	25–53
		Avg.± SD	78 ± 4	0	0	17 ± 4	192 ± 79	132 ± 58	4 ± 2	8 ± 1	17 ± 2	40 ± 11
	Avg.	--	105	2	9	25	316	165	15	57	11	98
W.R.	DS	Range	33–290	3–6	3–10	7–24	96–384	37–190	12–37	58–80	3–12	14–72
		Avg.± SD	98 ± 95	4 ± 1	8 ± 3	17 ± 7	253 ± 100	150 ± 59	29 ± 10	71 ± 9	9 ± 3	53 ± 22
	ER	Range	110–310	4–12	9–31	24–87	384–1536	100–430	24–67	100–198	9–24	80–540
		Avg.± SD	190 ± 83	8 ± 3	22 ± 7	50 ± 21	963 ± 425	295 ±114	52 ± 15	151 ± 39	19 ± 6	257 ± 152
	LR	Range	80–120	0	0	26–60	250–504	170–210	9–15	9–14	10–23	46–93
		Avg.± SD	95 ± 15	0	0	38 ± 12	376 ± 86	182 ± 16	12 ± 2	11 ± 2	18 ± 5	59 ± 18
	Avg.	--	128	4	10	35	530	209	31	77	150	123

Abbreviations: Avg. in the table denotes the average value. T.R. denotes Taipu River. W.R. denotes Wusong River. DS denotes the dry season. ER denotes the early rainy season. LR denotes the late rainy season.

**Table 2:** Comparison of the average heavy metal concentrations found in this study with concentrations found in other water bodies and with international water quality guidelines; units: ug/L.

Water Function	Rivers/lakes	Ba	Cd	Cr	Cu	Fe	Mn	Ni	Pb	V	Zn	References
Source of drinking water	Taipu River, China	105	2	9	25	316	165	15	57	11	98	this study
	Taihu Lake	--	--	--	4	34	41	--	17	--	0	[32]
	Rawal Lake, Pakistan (summer)	--	6	9	10	93	4	--	162	--	14	[34]
	Rawal Lake, Pakistan (winter)	--	25	97	17	76	13	--	223	--	22	[34]
	Rivers in Ghana	30	--	0.52	2.65	--	682	--	0.85	--	138	[33]
	Yangtze River in Nanjing Section, China	37	5	21	11	240	5	13	55	10	9	[36]
Industry	Wusong River, China	128	4	10	35	530	209	31	77	150	123	this study
	Lambro River, Italy	--	4.8	66	134	--	--	--	138.8	--	0	[38]
	Rivers in Latvia	--	0.02		0.56	--	3	0.34	0.1	--	3.35	[39]
	Ruda River, Polish	--	<3	<5	5–22	470–9610	179–1760	8–10	30–140	41–122	--	[28]
	DilDeresi (stream), Turkey	1200	7	30	31	1310	--	--	80	--	220	[40]
	Patancheru industrial area, India	78	--	18	--	162	73	26	2	99	--	[37]
	Hindon River, India	--	12	124	--	692	617	--	276	--	110	[41]
WHO drinking water guideline		--	3	50	2000	300	100	--	10	--	3000	[30]
USEPA drinking water standards		--	5	100	1300	300	50	--	15	--	5000	[31]

### 3.2. Risk Assessment

#### 3.2.1. Comprehensive Risk Indices for the Metal Pollution

The average CRIs for the rivers that were studied in the three seasons, at each of the 11 sampling sites, are presented in Table 3. The average values for all three seasons (the furthest right columns in Table 3) show that heavy metal pollution in the Taipu River posed low levels of risk, the CRI being 39.58, and that heavy metal pollution in the Wusong River posed moderate risks, the CRI being 68.76. The average CRI for the river affected strongly by industry, the Wusong River, was approximately two times higher than the CRI for the river that is protected so that it can be used to supply drinking water, the Taipu River. The integrated risks posed to the two rivers had similar seasonal distributions, and decreased in the order early rainy season > dry season > late rainy season. The highest CRIs were found in the early rainy season for both rivers, with moderate potential risks being posed in the Taipu River and high potential risks being posed in the Wusong River. Both rivers had low levels of risk posed by metal pollution in the late rainy season. The average risks were higher in the Wusong River than in the Taipu River in all three seasons. The greatest difference between the CRIs for the two rivers occurred in the early rainy season, and the difference between the CRIs for the rivers was smallest in the dry season (Table 3). 

**Table 3:** Comprehensive risk indices (CRIs) and potential integrated risks posed by metal pollution in the Taipu River (T.R.) and the Wusong River (W.R.).

Rivers	Sites	Dry Season		Early Rainy Season		Late Rainy Season		Seasonal Averagely	
		CRI	Risk Level	CRI	Risk Level	CRI	Risk Level	CRI	Risk Level
T.R.	T1	27.48	low	33.73	low	6.97	low	22.73	low
	T2	26.37	low	91.37	moderate	5.78	low	41.17	low
	T3	51.39	low	79.72	moderate	8.18	low	46.43	low
	T4	66.59	moderate	98.59	moderate	6.53	low	57.24	low
	T5	53.47	low	31.36	low	6.21	low	30.35	low
	**Avg.**	**45.06**	**low**	**66.95**	**moderate**	**6.73**	**low**	**39.58**	**low**
W.R.	W1	40.26	low	67.69	moderate	14.36	low	40.77	low
	W2	60.88	moderate	104.62	moderate	10.41	low	58.64	low
	W3	74.91	moderate	**142.97**	**high**	13.77	low	77.22	moderate
	W4	53.05	low	**123.32**	**high**	11.75	low	62.71	moderate
	W5	74.29	moderate	**136.90**	**high**	12.38	low	74.53	moderate
	W6	83.28	moderate	**193.13**	**high**	19.78	low	98.73	moderate
	**Avg.**	**64.44**	**moderate**	**128.11**	**high**	**13.74**	**low**	**68.76**	**moderate**

Table 3 shows that the risks in the middle and downstream sections of the Wusong River were higher than the risks in the upper section of the river in all three seasons, and that they reached high risk levels in the early rainy season at sites W3, W4, W5, and W6. The risks were slightly higher in the middle section than in the upstream and downstream sections of the Taipu River. The CRIs at all of the sampling sites except for site T5 decreased in the order: early rainy season > dry season > late rainy season. The different order for site T5 might have been caused by an unusual distribution and range of industries in the vicinity of that site, in the downstream section of the Taipu River (Figure 1). Of the 33 measurements we made, the highest CRI we found was at the entrance of a tributary into the Wusong River in the early rainy season. This site was strongly influenced by the urban district of Kunshan, which has high densities of both population and industry, and in which area the land is used intensively (Figure 1). The CRIs were higher than 100 at sites W3, W4, and W5 in the early rainy season. The spatial and seasonal variations indicate that, to more effectively prevent heavy metal pollution incidents, environmental monitoring and management measures should be focused on the middle and lower reaches of the Wusong River (the industrial river), especially at the beginning of the rainy season.

#### 3.2.2. Risks Posed by the Metals Analyzed

**Figure 2:**
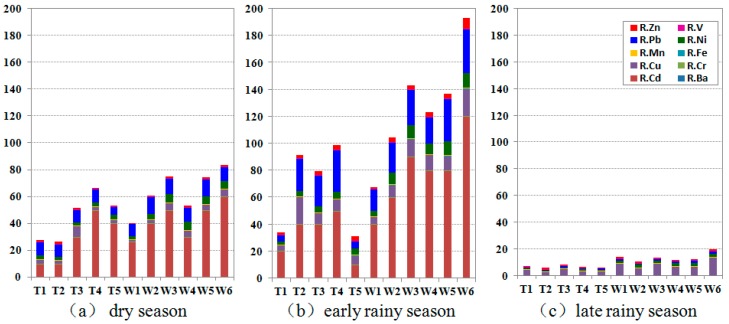
Accumulated column chart of risk ratios of the ten metals.

**Figure 3:**
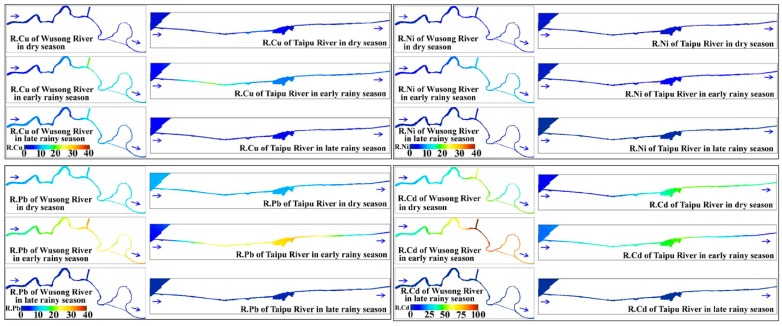
Seasonal and spatial distribution of pollution risk on Cu, Ni, Pb and Cd in the two rivers.

The accumulated risk ratios for the metals are plotted in Figure 2. The ratios are simply the contributions of the metals to the CRIs, and they vary seasonally and spatially. The risk ratios for Cd, Cu, Ni, and Pb dominated the total risk (Figure 2). The spatial distributions of the risks from these metals in the two rivers in each season are presented in Figure 3. The Cu, Ni, and Pb contributions to the CRIs decreased in the order Pb > Cu > Ni, and these three metals all posed low levels of risk in the two rivers in every season. The risks posed by these three metals had similar spatial and seasonal variabilities (Figure 3). The risk ratios were highest in the early rainy season and lowest in the late rainy season. The risks posed were clearly higher in the industrial area than in the area protected for use supplying drinking water. The risks posed were higher in the middle and downstream sections of the Wusong River than in the upstream sections. The highest level of risk in the Taipu River was found in the middle section.

We found that Cd was the most important of the ten metal pollutants, in terms of the risks posed. We found that the risks posed by Cd mostly dominated the CRIs in the dry and early rainy seasons. Similar conclusions were drawn in a study of Cd in the rivers flowing in and out of Taihu Lake, which included a risk assessment [17].The middle and downstream sections of the Taipu River and the Wusong River were at moderate risks from Cd pollution in the dry season, but the upstream sections of both rivers were at low potential risk from Cd (Figure 3). The risks posed by Cd in the early rainy season were low in the upstream and downstream sections of the Taipu River and moderate in the middle section. 

The upper section of the Wusong River had moderate risks posed by Cd pollution and the other sections had high risk levels. Cd was not detectable in the study area in the late rainy season, which might indicate that Cd in the surface water in the two rivers came mainly from anthropogenic sources rather than natural processes.

Ba, Cr, Fe, Mn, V, and Zn contributed little to the total risk posed by heavy metal pollution. The Ba, Cr, Mn, and V risk ratios were <1 in every sample, so these metals posed no potential risk to the aquatic ecosystems in the two rivers that were studied in any of the three seasons the samples were collected in, and they contributed less than 1% of the total risk posed by metal pollution [16]. Measurable potential risks were posed by Fe and Zn in some of the samples. Fe contributed 0.2%–4% of the CRIs. Fe contamination posed low levels of potential risk at sites W2 and W6 in the early rainy season, and no potential risk at the other sites or at other times. Zn contributed 0.8%–13% of the CRIs. Zn contamination posed low levels of potential risk at sites T1, T2, W3, and W6 in the dry season and at all of the sites in the early rainy season, and the highest risk ratio was found at the site in the tributary of the Wusong River in the early rainy season (Figure 2b).

It should be noted that the risks described in this study are relative risks, because the NOECs of the total metals in surface water were unavailable, because of a lack of toxicity data for the total metals, so the NOECs for the ionic species of the metals were used. These risk descriptions might, therefore, be overestimations of the actual risk levels. There is also some uncertainty about the toxicity coefficients for the heavy metals. The coefficients should be different for different species and for different types of ecosystem [22,42]. However it is difficult to obtain this information, so the same values were used for the two rivers that were studied, to simplify the assessment. These two aspects might be able to be improved upon in future research, and the assessment presented here should be seen as the most conservative possible when it is used in environmental risk management by decision makers.

### 3.3. Source Apportionment

A strong correlation (*P* < 0.05) was found between the concentrations of most of the heavy metals, from Pearson correlation analyses. The correlations were all verified as being significant at the 95% level using Bartlett’s tests of sphericity between the metal concentrations in the three seasons. PCA was, therefore, found to be appropriate for all three sets of data [7,22,28]. The source apportionment results are shown in Table 4.

#### 3.3.1. Possible Sources in Each of the Three Seasons

All of the heavy metal concentration variability (10 variables) in the dry season could be described using three principal components (Table 4) [26], and, together, these components (DPC1, DPC2, and DPC3) reflected 79.04% of the variability [7]. The loading values shown in Table 4 were classed as strong if they were >0.75, moderate if they were 0.75–0.50, or weak if they were 0.50–0.30 [7,43]. The source contribution from DPC1 was 43.199%, with strong loadings for Mn, Ni, and V and moderate loadings for Cd, Cr, Cu, and Pb.

**Table 4:** Cumulative contributions (%), loading values, and factor coefficients for the principal components for each season.

Items	Dry Season			Early Rainy Season			Late Rainy Season	
	DPC1	DPC2	DPC3	EPC1	EPC2	ERPC3	LPC1	LPC2
Cumulative Contribution								
**Metals**	43.199	67.483	79.042	71.162	81.318	89.630	60.778	79.827
Loading Values								
Ba	0.413	−0.014	**0.686**	**0.770**	−0.234	**0.562**	**0.857**	−0.123
Cd	**0.588**	−0.696	−0.169	**0.884**	0.024	−0.319	--	--
Cr	**0.665**	**0.608**	−0.247	**0.965**	−0.044	0.095	--	--
Cu	**0.644**	0.262	−0.538	0.673	**0.576**	0.121	**0.899**	−0.313
Fe	0.115	**0.922**	0.107	**0.903**	−0.279	−0.010	**0.866**	0.461
Mn	**0.900**	−0.265	−0.179	**0.837**	0.357	0.090	**0.750**	0.416
Ni	**0.900**	−0.225	0.052	**0.890**	−0.229	−0.310	**0.893**	0.206
Pb	**0.656**	−0.031	0.495	**0.779**	0.416	0.325	**0.897**	−0.252
V	**0.900**	−0.002	0.090	**0.864**	−0.442	−0.202	-0.150	**0.931**
Zn	0.273	**0.730**	0.088	**0.836**	0.035	0.374	**0.618**	−0.230
Factors’ Coefficients								
T1	−1.823	**2.587**	0.694	−3.740	−0.702	**0.615**	−2.836	−0.564
T2	−1.552	**2.257**	0.708	−0.459	**2.030**	0.413	−1.611	−0.602
T3	0.304	**1.531**	−1.718	−1.120	**0.583**	0.251	−0.773	**1.168**
T4	−0.928	−1.437	−0.127	−0.279	**1.182**	−0.001	−1.793	−0.070
T5	−1.476	−0.814	−0.816	−2.293	−0.643	**1.207**	−2.875	−0.991
W1	−3.780	−2.427	−0.200	−3.174	−0.345	−0.794	**1.339**	−1.208
W2	0.590	0.034	**0.953**	**2.378**	−1.749	0.593	**1.459**	0.324
W3	**2.695**	−0.015	−0.347	**1.501**	−0.195	−1.286	0.512	**1.274**
W4	**1.603**	0.040	−0.430	**0.052**	−0.138	−0.769	0.259	**0.873**
W5	**2.594**	−0.986	2.076	**1.820**	−0.257	−1.346	**2.240**	1.927
W6	**1.773**	−0.769	−1.193	**5.314**	0.233	1.118	**4.079**	−2.131

Therefore, DPC1 could be concluded as being the dominant source of Mn, Ni, and V, and to partly explain the sources of Cd, Cu, Cr, and Pb. These metals are typically industrial pollutants, so DPC1 could be ascribed to pollutant sources from the dyeing, electroplating, fertilizer production, leather, paint, and printing industries [37]. The contribution made by DPC2 to the sources was 24.283%, and it had a strong loading for Fe and moderate loadings for Zn and Cr. The most plausible source that DPC2 was associated with was metals released from the earth, so geological weathering could be assumed to be the DPC2 source, and Fe in the rivers might have originated mainly from pedogenic processes [7]. DPC3 contributed 11.559% of the sources, with a moderate loading for Ba and a weak loading for Pb. Ba can come from the earth itself [7], but the simultaneous release of Ba and Pb is typical of metals in traffic pollution [44]. There is no evidence that the background Ba concentrations in the Taihu basin are particularly high, but there are many busy roads crisscrossing the region studied (Figure 1). The DPC3 source in the dry season was, therefore, assumed to be traffic pollution.

Three principal components (EPC1, EPC2, and EPC3) were also identified in the early rainy season. EPC1 dominated the sources of most of the metals, the contribution of EPC1 to the total sources being 71.162%, with strong loadings for Cr, Ba, Cd, Fe, Mn, Ni, Pb, V, and Zn and a moderate loading for Cu. Industrial sewage is indicated by Cd, Cr, Ni, Pb, V, and Zn, and Fe and Mn could be attributed to geochemical sources that cause them to be found in the storm water that flows into the rivers in the rainy season [7,45]. EPC1 could, therefore, be assumed to include the joint sources of storm water runoff and industrial emissions in the early rainy season. EPC2 had a moderate loading for Cu. Cu pollution mainly originates from machinery manufacturing processes, especially from such industries as copper processing and iron and steel production [46]. These industries have been encouraged by the Chinese government, and are listed as such in the industrial structural adjustment guidance announced by the National Development and Reform Commission [47]. These industries have led the industrial development of the Taihu basin. EPC3 had a moderate loading for Ba and weak loadings for Pb and Zn. This component had the characteristics of traffic pollution [44]. Traffic pollutants will enter surface water with atmospheric wet deposition in the rainy season [48]. EPC3 was therefore assumed to be atmospheric wet deposition.

Two principal components (LPC1 and LPC2) were found in the late rainy season. LPC1 had strong loadings for Ba, Cu, Fe, Mn, Ni, and Pb, and a moderate loading for Zn, and it was estimated that, like EPC1, this component indicated joint sources, storm water runoff and industrial emissions. LPC2 had a strong loading for V, and the source indicated could have been industrial emissions [7,45].

#### 3.3.2. Dominant Sources in Different Samples

There are a number of possible sources of the heavy metals found in the rivers that were studied, and they could be determined and traced using the factor coefficients [49,50]. The factor with the highest coefficient could potentially be associated with the dominant source, and these are shown in bold in Table 4. If all of the coefficients are negative the metal concentrations can be regarded as being quite low, and it can be assumed that they can be neglected.

In the dry season, the metals in the upstream section of the Taipu River (sites T1–T3) were mainly from geological weathering (DPC2) and the metals in the downstream section (sites T4 and T5) had rather low concentrations (Table 4). The Taipu River is therefore rarely influenced by anthropogenic activities in the dry season. This indicates that risks posed by heavy metals in the area that is protected for use supplying drinking water mainly originated from pollution accumulated over a long period and the environmental background metal concentrations in the region in the dry season. The river was mainly affected by emissions from mechanical manufacturing industries (EPC2) in the early rainy season. The metal concentrations in the late rainy season were so low that they were all negligible, except for at site T3, which might have been affected by industrial emissions (LPC2).

The metal loads in the upper reaches of the Wusong River were rather low, and the coefficients at site W1 were all negative in the dry and early rainy seasons. The heavy metals at site W2 in the dry season came mainly from traffic pollution near the site(DPC3) and the metals at the other sites came from industrial emissions (DPC1) (Table 4). The joint dominant heavy metal sources in the river in the rainy season were storm water runoff and industrial discharges (EPC1 and LPC1). The heavy metal sources to the Wusong River for the dry and rainy seasons were completely consistent with the dense distribution of industrial sites in this area.

## 4. Conclusions

The density of the population and the number of industrial sites in the Taihu basin are likely to negatively influence the surface waters in the area, both in the industrial areas and in the area that is protected so that it can be used to supply drinking water. There were significant differences between the heavy metal concentrations in the two rivers that we studied. The heavy metal concentrations in the industrial area (the Wusong River) were 1.2–2.0 times higher than the concentrations in the protected area. The metal concentrations in both rivers decreased in the order early rainy season > dry season > late rainy season. 

The heavy metal concentrations were found, on average, to pose low levels of risk in the Taipu River and moderate risks in the Wusong River. Ba, Cr, Mn, and V posed no potential risks in both rivers, and Fe and Zn posed low risk levels at some sites. The Cd, Cu, Ni, and Pb contributions to the total risk levels were dominant. Cu, Ni, and Pb posed low levels of risk in every season and Cd, which posed high levels of risk, was the most important pollutant, in terms of risk, of the ten metals that were analyzed.

The spatial and seasonal variations in the potential hazards posed by the metals were assessed, and the risks posed were higher in the middle and lower reaches of the Wusong River than in the upstream section of the river, and the highest risks posed in the Taipu River were in the middle section. The risks posed by the metals were highest in the early rainy season and next highest in the dry season; the risks in the late rainy season were the lowest. Therefore, to more effectively prevent incidences associated with heavy metal pollution, attention should be focused on the middle and lower reaches of the polluted industrial river (the Wusong River), especially at the beginning of the rainy season. 

Metals in the Taipu River in the dry season probably originated mainly from pollution accumulated over long periods and the background concentrations in the environment in the basin. The main sources in the rainy season were emissions from mechanical manufacturing industries. The metals in the Wusong River predominantly came from industrial emissions, and the dense distribution of industrial sites in this area has caused heavy metal pollution to pose high levels of risk to the aquatic ecosystem, especially in the early rainy season.
